# Physical Parameters in Young Competitive Padel Players: Strength, Power, Agility, and Smash Velocity Assessments

**DOI:** 10.3390/sports13040104

**Published:** 2025-03-28

**Authors:** Bernardino J. Sánchez-Alcaraz, Iván Martín-Miguel, Rafael Conde-Ripoll, Diego Muñoz, Adrián Escudero-Tena, Alejandro Sánchez-Pay

**Affiliations:** 1 Faculty of Sport Sciences, University of Murcia, 30700 Murcia, Spain; aspay@um.es; 2 Faculty of Sport Sciences, University of Extremadura, 10003 Cáceres, Spain; ivanmartinmiguel97@gmail.com (I.M.-M.); diegomun@unex.es (D.M.); adescuder@alumnos.unex.es (A.E.-T.); 3 Department of Sports Sciences, Faculty of Medicine, Health and Sports, Universidad Europea de Madrid, 28670 Madrid, Spain; info.conderipoll@gmail.com

**Keywords:** racket sports, padel, adolescents, conditioning, training

## Abstract

The main objective of this study was to analyze the physical fitness parameters of young competitive padel players, compare potential differences between male and female players, and examine the relationships among various physical fitness variables in this population. The sample consisted of 18 players (10 boys and 8 girls) aged between 12 and 16 years old belonging to the Technification program for minors of the Valencian Padel Federation. The players completed a test battery that consisted of different tests: CMJ jump, internal and external shoulder rotator strength, manual dynamometry, functional upper body strength (forehand throw, backhand throw, bilateral overhead throw, and serve throw), smash speed, 5 × 10 m agility test, and tapas test. Data analysis was carried out with SPSS software for Windows (Version 25.0, IBM Corp., Armonk, NY, USA). The results showed that the boys obtained significantly higher values in the tests of dynamometry, speed, agility (tapas test), and throws (forehand, backhand, serve, and over the head), and the girls obtained significantly higher values in the test of shoulder external rotation (non-dominant). At the same time, the force variables were significantly and positively related to each other. The CMJ values are also significantly and positively correlated with the variables of shoulder rotation, sprint speed, and medicine ball throws. Regarding agility, significant and positive correlations were found in the tapas test. However, the 5 × 10 m test showed negative and significant correlations with some variables. It has been observed that the results obtained coincide with the results found in other studies carried out with players of the same age in other sports, such as tennis and soccer.

## 1. Introduction

Padel is a doubles racket sport played on a 20 × 10-m enclosed court with a central net and perimeter walls of glass or metal mesh (3–4 m high) that allow ball rebounds as part of the game [1]. Its rapid global expansion over the past decade, with federations in 150 countries and Spain leading in participation [1,2], is driven by its social and recreational appeal, ease of learning, and longer rallies compared to other racket sports [3,4,5,6].

Padel is characterized by intermittent bouts of moderate-to-high intensity effort [7,8] and imposes a unique match load on players, differing from singles tennis and more closely resembling doubles tennis [9]. Despite this, it is considered predominantly an aerobic sport [10]. Studies on adult players’ movements indicate they cover 2000–3000 m per match, with approximately 50% of this distance occurring during active play [11,12], with a player load of ~50 arbitrary units [13]. These values vary depending on match competitiveness, temporal factors, and player level [14]. On average, players cover 11 m per point and around 80 m per game [12]. Male adult players spend over 50% of the match moving at speeds below 3 km·h⁻^1^, with roughly 30% at speeds between 3 and 6 km·h⁻^1^ [11,15]. Professional male padel players predominantly perform lateral and forward movements, frequently executing jumps for split-steps, smashes, and rotations [16].

Game actions and temporal aspects in padel vary according to players’ age, gender, and skill level [17,18,19], which may also influence physical demands. While padel players perform numerous short, high-intensity movements due to the frequent ball exchanges and the court’s limited dimensions [20], they must also execute pivots and rotations before striking the ball, given the presence of walls that allow for rebounds [21]. Consequently, strength, movement speed, and change-of-direction ability play a crucial role in padel performance.

Several studies have focused on examining physical parameters in padel players, ranging from junior to professional [22,23], including amateur players [24,25]. Regarding physical parameters in junior padel players, research has highlighted improvements in strength, power, and agility over a competitive season, with youth players demonstrating significant gains in isometric squat performance, jump height, and core strength after 20 weeks of training [26]. Differences based on gender have also been examined in several studies. Pradas et al. [27] have shown that male players exhibit superior grip strength, vertical jump power, and lateral movement, while female players excel in reaction time and flexibility. These values may be attributable to the biological changes that occur at puberty [28]. Sánchez-Alcaraz et al. [29] have concluded that males achieve faster sprint times and greater upper-body force. In contrast, Courel-Ibáñez and Llorca Miralles [30] have observed that both genders display similar overall fitness levels, with males tending to outperform females in jumping ability, and greater padel experience appears to enhance upper-limb throwing strength. Additionally, younger players show lower values in flexibility, gesture speed, and cardiorespiratory capacity compared to older junior players, underscoring the impact of physical maturation [27].

A better understanding of the physical profile of competitive players is also essential for improving the padel training process and talent scoping [31,32]. Information about how players respond to particular tests assists coaches in setting benchmarks and developing effective training drills accordingly [33]. However, information about fitness determinants in young padel players is very limited [34]. Similarly, another gap exists regarding research on physical fitness parameters, representing a limitation in this field [35]. The objective of this study was to analyze the physical fitness parameters of young competitive padel players, compare potential differences between male and female players, and examine the relationships among various physical fitness variables in this population.

## 2. Materials and Methods

### 2.1. Participants

The participants were 18 padel players (10 boys and 8 girls) aged between 12 and 16. The players belonged to the Valencian Padel Federation’s under-age training program. Table 1 shows the characteristics of the participants in the study.

### 2.2. Procedure

First, informed consent was obtained from the Valencian Padel Federation, the sports club where the assessments took place, and the study participants. Data collection was conducted in a single morning session, between 10:00 and 14:00, at an outdoor facility with a temperature of 12 °C. After a standardized five-minute warm-up, players completed a series of physical tests organized into four stations: (a) on-court smash test, (b) medicine ball throw test (forehand, backhand, bilateral overhead, and shot-put), (c) strength tests (handgrip dynamometry, countermovement jump, and internal and external shoulder rotators), and (d) agility tests (5 × 10 shuttle run with changes on the right and left leg, and the tap test). Players were divided into four groups, each assigned to a station, rotating every five minutes with a five-minute rest between stations. Ethical approval for this study was obtained from the Ethics Committee of the University of Extremadura (166//2023).

### 2.3. Instruments

Various instruments were selected to assess the physical performance of padel players and smash speed, following methodologies from similar research in padel and tennis [36,37,38].

Countermovement Jump (CMJ): Jump height was measured using a Chronojump contact platform (Chronojump, Barcelona, Spain) and the Chronojump software version 1.7.1.8 for MAC. Participants started from an upright position with hands on their hips and feet shoulder-width apart. Each participant performed two attempts, with a two-minute rest between jumps. The best attempt, measured in centimeters, was recorded for analysis.

Internal and External Shoulder Rotator Strength: A strain gauge was used to assess shoulder rotator strength. Participants lay in a supine position with the elbow flexed at 90° and the shoulder abducted at 90°, keeping the wrist aligned with the forearm. Once positioned, they performed an internal rotation first, returned to the initial position, and then executed an external rotation, repeating the process with the opposite shoulder. Each test was performed twice per shoulder, with rest periods between attempts.

Handgrip Dynamometry: This test assessed the maximal isometric strength of the finger flexors using a Smedley III T-18A dynamometer (Takei, Tokyo, Japan), which has a measurement range of 0–100 kg with 0.5 kg increments and an accuracy of ±2 kg. The test was conducted with the arm extended and positioned close to the torso without making contact. Participants exerted maximal force for three seconds. After a familiarization phase with submaximal repetitions, each participant performed two maximal attempts with a two-minute rest between trials. The best result, measured in kilograms, was recorded for analysis.

Upper-body functional strength: Upper-body explosive functional strength was assessed through three medicine ball (2 kg) throw tests, simulating forehand, backhand, overhead bilateral, and serve motions. Participants stood behind the throwing line, with a 15 m measuring tape placed perpendicular to it on the ground. Two evaluators marked the ball’s landing zone, recording distances in 0.10 m increments. Each throw type was performed twice, with a two-minute rest between attempts. The best attempt, measured in meters, was recorded for analysis.

Forehand/backhand throw: The participant stood sideways to the throwing direction, holding the ball with both hands on the side of the body corresponding to the throwing motion (forehand or backhand). The dominant hand generated force, while the non-dominant hand guided the movement. At the evaluator’s signal, the participant threw the ball as far as possible.

Overhead bilateral throw: The participant faced the throwing direction, holding the ball with both hands behind the head. At the evaluator’s signal, the participant executed an explosive motion, throwing the ball overhead in a manner similar to a soccer throw-in.

Shot-put throw: The participant stood sideways to the throwing direction, holding the ball in the palm of the dominant hand beside the head. At the evaluator’s signal, they performed an explosive motion, throwing the ball similarly to a shot-put technique.

Smash velocity: The velocity of the shot was gauged using the speed radar, designated the ‘Pocket Radar Ball Coach’, manufactured by Technology Sport. The player was asked to hit 8 smashes, 2 m from the net. The radar was placed behind the player at the same hitting height and facing the direction of the ball. The radar measures with a speed range between 40 and 210 km/h and is a valid and highly sensitive tool for ball velocity measurement (CITA). For the analysis, the value of the 6 best shots in km/h were counted.

The 5 × 10 agility test: This test assessed change-of-direction ability, speed, and agility. Participants started behind the starting line in an upright position, facing a line 5 m away. At the evaluator’s signal, they sprinted to the 5 m line, stepping on it with one foot before making a 180° turn to sprint back to the starting line. The test concluded when the participant crossed the starting line. Each player performed the test twice using their dominant leg for the turn and twice with their non-dominant leg. The faster time of the two attempts for each leg was recorded for analysis.

**Figure 1:**
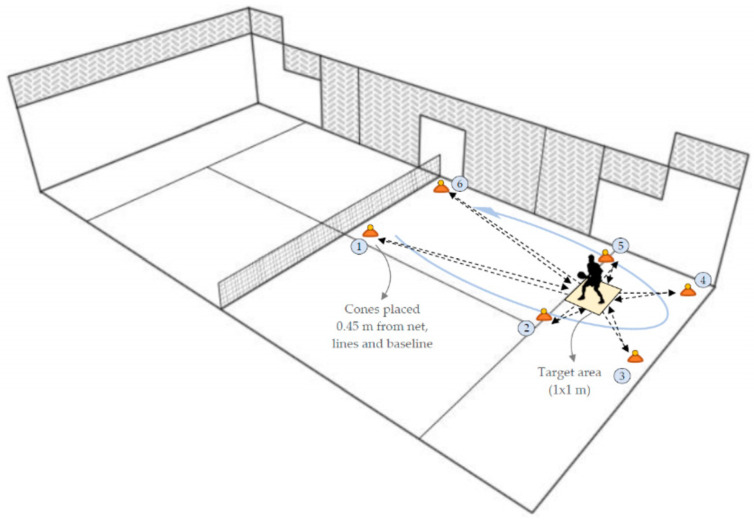
Tapas 6R test.

Tapas 6R test: This padel-specific agility test, originally named the Tapas 6R Test (Figure 1), was designed to evaluate padel players’ agility. Six balls were placed on flat cones in specific positions: 0.45 m from the lines, walls, net, and baseline. Players started at the center of the service line, within a designated 1 × 1 m target area where they had to place the collected balls. They followed a set sequence, sprinting to each position, picking up a ball, turning, returning to the target area, and placing the ball without dropping it. Players used their dominant hand for both retrieval and placement. The test ended when the last ball was placed in the target area.

### 2.4. Statistical Analysis

A descriptive analysis was conducted, including the calculation of mean and standard deviation (M ± SD) for the studied variables. Given the small sample size, the Shapiro–Wilk and Levene tests were used to assess normality and homogeneity of variances for each variable. A Student’s *t*-test was applied to compare mean differences between genders (male and female). Correlation analysis was performed using Pearson’s *r* coefficient to examine relationships between different tests and participants’ ages. Correlation values were classified as Trivial (0–0.1), Small (0.1–0.3), Moderate (0.3–0.5), Large (0.5–0.7), Very Large (0.7–0.9), Nearly Perfect (0.9), and Perfect (1.0) [39]. Statistical significance was set at *p* < 0.05. All analyses were conducted using SPSS for Windows (Version 25.0, IBM Corp., Armonk, NY, USA).

## 3. Results

Table 2 presents the descriptive data of the studied variables based on participants’ gender. Significant differences were found, with male players exhibiting higher values in dynamometry (both dominant and non-dominant hand), speed (average and maximum), agility (Tapas test), and throwing performance (forehand, backhand, serve, and overhead). In contrast, female players showed superior performance in the non-dominant shoulder external rotation test.

Table 3 presents the correlation results between the different variables under study. As observed, strength-related variables showed significant positive correlations among themselves. Specifically, handgrip dynamometry values for both the dominant and non-dominant sides correlated significantly and positively with countermovement jump performance, shoulder rotation strength (dominant, non-dominant, internal, and external), smash speed, and medicine ball throws (forehand, backhand, bilateral overhead, and serve). Countermovement jump values also exhibited significant positive correlations with shoulder rotation strength (dominant and non-dominant, internal and external), smash speed, and medicine ball throws (forehand, backhand, bilateral overhead, and serve).

Regarding agility variables, significant positive correlations were found with the Tapas agility test. However, the 5 × 10 agility test (changes of direction with both the left and right legs) showed significant negative correlations with certain strength variables, such as handgrip dynamometry, medicine ball throws, and shoulder rotation strength.

## 4. Discussion

The main objective of this research was to analyze the physical fitness parameters of young competitive padel players, compare potential differences between male and female players, and examine the relationships among various physical fitness variables in this population.

First, regarding strength-related variables, boys obtained higher results in both the handgrip dynamometry test and all four types of medicine ball throws than women players. These findings align with previous studies, such as that of Courel-Ibáñez and Llorca-Miralles [30], which analyzed the physical fitness levels of young competitive padel players in Andalusia [30]. The average medicine ball throw distances recorded in this study for both male and female players were higher than those reported by Sánchez-Alcaraz et al. [29]. These differences may be attributed to the varying skill levels of the padel players in both studies. On the other hand, regarding the countermovement jump test, no significant differences were found between boys and girls. This contrasts with the findings of Courel-Ibáñez and Llorca-Miralles [30], where boys achieved significantly higher values in this variable.

Regarding agility, the results were similar to those reported by Sánchez-Alcaraz et al. [40] in 12- and 13-year-old soccer players but showed faster times compared to the study by Sánchez-Alcaraz et al. [41], which evaluated 24 tennis players aged 8 to 10 years. These differences may be attributed to the age variation among participants [41]. The padel-specific agility test, known as Tapas, revealed significantly better times for boys compared to girls, aligning with the findings of Courel-Ibáñez and Llorca-Miralles [30]. Both agility tests (5 × 10 and Tapas) showed a significant positive correlation, suggesting that this new test could be a valuable tool for assessing agility levels in youth padel players within development programs. However, the 5 × 10 m agility test showed a negative correlation with strength variables. These results could be related because padel is characterized by a high rhythm but lower intensity compared to similar racket sports, especially in young padel players [30,42].

Regarding smash velocity, boys exhibited higher average and maximum speeds than girls. These findings are particularly relevant since the smash is considered the most decisive shot in padel, being the technical–tactical action that generates the highest number of winning points in amateur and professional padel players [43]. Additionally, smash speed correlated positively and significantly with other physical tests, such as handgrip dynamometry, countermovement jump (CMJ), and medicine ball throws. Other studies have also reported positive correlations between strength, measured through medicine ball throws, and serve speed in young tennis players [44,45]. Similarly, Sánchez-Pay et al. [38] found that higher medicine ball throw results were associated with greater serve speed in wheelchair tennis players. Conversely, some studies suggest that strength and power values do not necessarily correlate with tennis serve speed [37]. Additionally, Hayes et al. [46] observed a positive correlation between CMJ and tennis serve speed, while Fett et al. [45], in a study with 1016 tennis players (625 boys and 394 girls) aged between 9.4 and 17.9 years, found no correlation between CMJ and tennis serve speed. It is important to highlight the role of the technique on the velocity of the smash, so the differences between the mentioned studies could be related to different players’ levels and skills in shot technique and biomechanics. Future research should consider this variable when studying the speed and accuracy of the shot.

This study presents several limitations that should be considered. First, the small sample size limits the generalizability of the results. Additionally, player dominance (left-handed vs. right-handed) was not taken into account, suggesting that future studies should increase the sample size and compare not only by gender but also by other variables such as age, playing level, laterality, court position, etc. Regarding the physical tests, from a biomechanical perspective, no anthropometric measurements of body segments and levers were taken, which could influence the results of the medicine ball throws and smash speed tests. Furthermore, smash speed was assessed through an analytical field test rather than within a competitive match setting. Finally, these results can only be applied to high-performance young players, and it would be interesting to replicate the methodology of this study at different levels of competition and age.

The findings of this study have important practical applications for training design based on players’ physical and technical characteristics. Given the strong relationship between medicine ball throw (MBT) performance and smash speed in padel, it is recommended to incorporate MBT exercises (with and without displacement, with and without jumping, overhead, etc.) into physical conditioning programs to enhance smash performance.

## 5. Conclusions

In conclusion, the findings of this study provide a detailed understanding of the physical performance differences between young padel players based on gender, as well as the relationships among various physical fitness parameters. The results confirmed that male players exhibited higher values in strength, speed, and agility tests, whereas female players demonstrated superior performance in the non-dominant shoulder external rotation test. These differences may be attributed to physiological and biomechanical factors inherent to each gender.

Additionally, the positive correlations between handgrip strength, countermovement jump performance, and smash speed suggest that the development of both upper- and lower-body strength is crucial for enhancing padel performance. In particular, medicine ball throw performance emerged as a strong predictor of smash speed, highlighting its relevance in training programs.

## Figures and Tables

**Table 1:** Participants characteristics.

	Male M (S.D.)	Female M (S.D.)
Number	10	8
Age	13.50 (1.95)	14.00 (1.22)
Weight (kg)	54.33 (15.16)	56.50 (8.86)
Weekly hours of training	4.35 (1.63)	3.70 (1.09)
Experience in padel (ages)	6.20 (1.61)	7.20 (3.11)
Laterality	8 right-handed/2 left-handed	5 right-handed/0 left-handed

Note: M = Mean, S.D. = Standard deviation.

**Table 2:** Mean values and standard deviation by gender.

	Male M (SD)	Female M (SD)	F	*p*-Value
HD D (kg)	29.42 (11.19)	29.00 (4.59)	9.812	0.008 **
HD ND (kg)	24.35 (8.88)	23.65 (3.47)	32.898	0.000 **
CMJ (cm)	27.82 (5.62)	27.51 (4.10)	1.26	0.280
AG R (s)	2.85 (0.12)	2.83 (0.10)	0.392	0.542
AG L (s)	2.77 (0.27)	2.90 (0.13)	0.760	0.399
IR D (kg)	80.01 (19.86)	97.10 (21.05)	0.000	0.984
ER D (kg)	78.27 (26.35)	94.14 (20.76)	0.246	0.628
IR ND (kg)	72.98 (25.99)	76.81 (15.33)	2.376	0.147
ER ND (kg)	74.64 (27.00)	80.91 (10.30)	8.459	0.012 *
Mean V (km/h)	112.17 (13.39)	103.63 (5.20)	8.143	0.021 *
Max V (km/h)	127.50 (12.25)	107.80 (6.21)	7.652	0.017 *
AG TAP (s)	18.84 (1.04)	19.12 (0.67)	8.012	0.014 *
MBT Fh (m)	8.65 (2.49)	7.48 (0.99)	8.083	0.014 *
MBT Bh (m)	8.10 (2.48)	6.69 (0.60)	11.266	0.004 **
MBT BI (m)	6.22 (1.79)	6.16 (0.92)	10.540	0.006 *
MBT S (m)	8.00 (2.25)	7.41 (0.88)	10.904	0.006 *

Note: M: Mean, SD: Standard deviation, *: *p* < 0.005, **: *p* < 0.001, HD: Handgrip dynamometry (D: Dominant; ND: Non-dominant), CMJ: Countermovement jump, AG = Agility (R: Right leg; L = Left leg), IR: Shoulder internal rotation (D: Dominant; ND: Non-dominant), ER: External shoulder rotation (D: Dominant; ND: Non-dominant), V: Velocity; AG TAP: Agility tapas; MBT: Medicine ball throw (Fh: Forehand, Bh: Backhand, BI: Bilateral overhead, S: Shot-put).

**Table 3:** Pearson correlation between different variables analyzed.

Variable	HD D	HD ND	CMJ	AG R	AG L	IR D	ER D	IR ND	ER ND	V	AG TAP	MBT Fh	MBT Bh	MBT BI	MBT S
HD D (Kg)	—														
HD ND (kg)	0.965 **	—													
CMJ (cm)	0.763 **	0.808 **	—												
AG R	−0.610 *	−0.607	−0.399	—											
AG L	−0.542 *	−0.653 **	−0.517 *	0.393	—										
IR D	0.749 **	0.684 **	0.725 **	−0.488	−0.120	—									
ER D	0.846 **	0.762 **	0.626 *	−0.540 *	−0.336	0.860 **	—								
IR ND	0.878 **	0.828 **	0.616 *	−0.559 *	−0.535 *	0.732 **	0.940 **	—							
ER ND	0.889 **	0.875 **	0.657 **	−0.535 *	−0.607 *	0.714 **	0.890 **	0.963 **	—						
V (km/h)	0.751 *	0.777 **	0.771 **	−0.372	−0.523	0.420	0.183	0.369	0.544	—					
AG TAP	−0.703 **	−0.811 **	−0.676 **	0.532 *	0.599 *	−0.384	−0.411	−0.542 *	−0.630 *	−0.829 **					
MBT Fh (m)	0.762 **	0.788 **	0.737 **	−0.529 *	−0.341	0.642 **	0.513	0.588 *	0.639 *	0.925 **	−0.742 **	—			
MBT Bh (m)	674 **	0.757 **	0.673 **	−0.522 *	−0.403	0.433	0.366	0.480	0.558 *	0.893 **	−0.833 **	0.926 **	—		
MBT BI (m)	0.872 **	0.859 **	0.817 **	−0.532 *	−0.358	0.812 **	0.693 **	0.687 **	0.735 **	0.900 **	−0.743 **	0.915 **	0.785 **	—	
MBT S (m)	0.853 **	0.903 **	0.831 **	−0.474	−0.535 *	0.662 **	0.650 **	0.709 **	0.793 **	0.883 **	−0.720 **	0.889 **	0.835 **	0.868 **	—

Nota: HD: Handgrip dynamometry (D: Dominant; ND: Non-dominant), CMJ: Countermovement jump, AG = Agility (R: Right leg; L = Left leg), IR: Shoulder internal rotation (D: Dominant; ND: Non-dominant), ER: External shoulder rotation (D: Dominant; ND: Non-dominant), V: Velocity; AG TAP: Agility tapas; MBT: Medicine ball throw (Fh: Forehand, Bh: Backhand, BI: Bilateral overhead, S: Shot-put).* *p* <0.05; ** *p* < 0.01

## Data Availability

The original contributions presented in the study are included in the article; further inquiries can be directed to the corresponding author.
